# Transverse Carpal Ligament and Forearm Fascia Release for the Treatment of Carpal Tunnel Syndrome Change the Entrance Angle of Flexor Tendons to the A1 Pulley: The Relationship between Carpal Tunnel Surgery and Trigger Finger Occurence

**DOI:** 10.1155/2013/630617

**Published:** 2013-06-26

**Authors:** Nazım Karalezli, Harun Kütahya, Ali Güleç, Serdar Toker, Hakan Karabörk, Tunc C. Ogun

**Affiliations:** ^1^Department of Orthopaedics and Traumatology, Necmettin Erbakan University Meram School of Medicine, Konya, Turkey; ^2^Department of Orthopaedics and Traumatology, Konya Beyhekim State Hospital, 42100 Selçuklu, Konya, Turkey; ^3^Department of Orthopaedics and Traumatology, Konya Meram Medical Training and Research Hospital, Konya, Turkey; ^4^Geodesy and Photogrammetry Engineering Department, Engineering and Architecture Faculty, Selçuk University, Konya, Turkey; ^5^Private Konya Medicana Hospital, Department of Orthopaedics and Traumatology, Konya, Turkey

## Abstract

*Purpose*. The appearance of trigger finger after decompression of the carpal tunnel without a preexisting symptom has been reported in a few articles. Although, the cause is not clear yet, the loss of pulley action of the transverse carpal ligament has been accused mostly. In this study, we planned a biomechanical approach to fresh cadavers. *Methods*. The study was performed on 10 fresh amputees of the arm. The angles were measured with (1) the transverse carpal ligament and the distal forearm fascia intact, (2) only the transverse carpal ligament incised, (3) the distal forearm fascia incised to the point 3 cm proximal from the most proximal part of the transverse carpal ligament in addition to the transverse carpal ligament. The changes between the angles produced at all three conditions were compared to each other. *Results*. We saw that the entrance angle increased in all of five fingers in an increasing manner from procedure 1 to 3, and it was seen that the maximal increase is detected in the middle finger from procedure 1 to procedure 2 and the minimal increase is detected in little finger. *Discussion*. Our results support that transverse carpal ligament and forearm fascia release may be a predisposing factor for the development of trigger finger by the effect of changing the enterance angle to the A1 pulley and consequently increase the friction in this anatomic area. *Clinical Relevance*. This study is a cadaveric study which is directly investigating the effect of a transverse carpal ligament release on the enterance angle of flexor tendons to A1 pulleys in the hand.

## 1. Introduction

Carpal tunnel syndrome (CTS) is the most common upper limb entrapment neuropathy [1] in which patients suffer from numbness and pain in the hand and digits. The prevalence is 2,7% [2]. Trigger finger (TF) is also a common clinical disorder characterised by painful locking of the involved digit. Both disorders are causes of disability and they often coexist in the same hand [3, 4]. The relationship between the two conditions and their exact cause is not clear [5]. The appearance of TF after decompression of the carpal tunnel without a preexisting symptom has also been reported in a few papers. According to these reports, in some of patients, the onset of the TF occurred within few months of open carpal tunnel release [6]. Although the cause is also not clear yet, the loss of pulley action of the transverse carpal ligament (TCL) has been accused mostly. Focusing on the condition, an argument arised: could the loss of pulley function of the TCL pave the way for the transfer of forces to the next pulley resulting in a change in the entrance angle of the flexor tendon into the A1 pulley? In order to be able to answer this question, a biomechanical study on fresh cadavers was planned and reported in this study.

## 2. Materials and Methods

**Figure 1:**
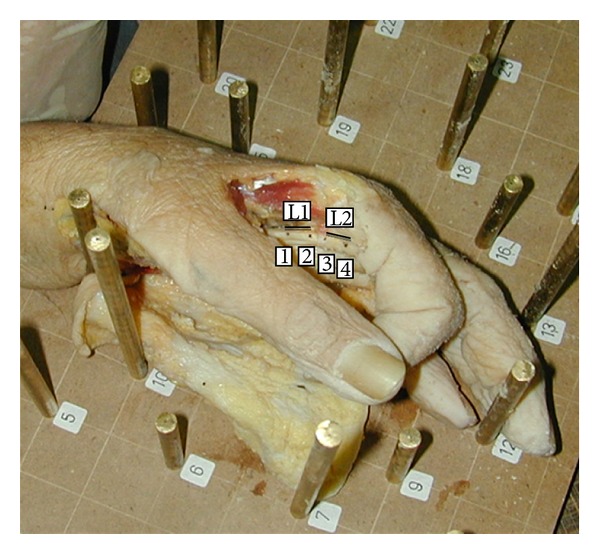
Four points were marked on the flexor tendon consecutively using a surgical pen and two lines were created as line 1 (points 1 and 2) and line 2 (points 3 and 4). L1: line 1, L2: line 2.

**Figure 2:**
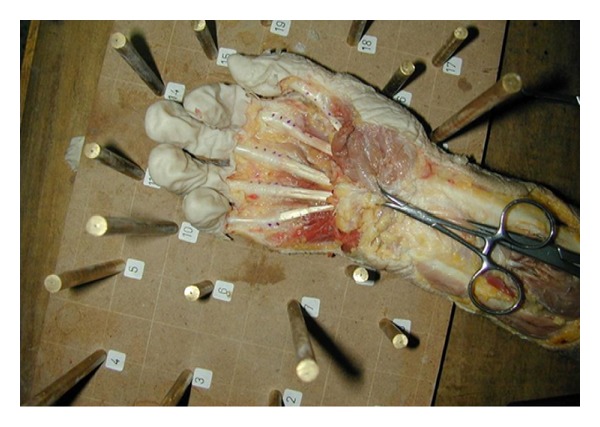
The photogrammetric evaluation was performed using 8–10 control points and 6 tie points.

**Figure 3:**
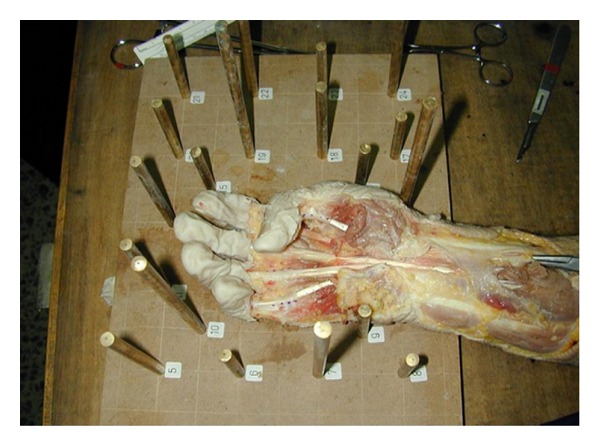
A 1 cm proximal excursion of the first point on the flexor tendon proximal to the A1 pulley was provided by the proximal pull of both finger flexors or thumb flexor of one finger at a time.

The study was performed on 10 fresh amputees of the arm. The overlying skin from the distal third of the forearm to the proximal interphalangeal joints of all the fingers and the interphalangeal joint of the thumb was resected. The hand was placed on the table with extension at the interphalangeal and metacarpophalangeal joints. Four points were marked on the flexor tendon consecutively using a surgical pen and two lines were created as line 1 (points 1 and 2) and line 2 (points 3 and 4) (Figure 1). Images from various projection centers were taken providing overlapping areas, using a nonmetric digital camera (Nikon Coolpix 950) which was calibrated before the process. In the outer projection step of photogrammetric restitution, 3D reference frame with target points whose coordinates in space were known was used. The Pictran software (Pictran-D and B modules) was used for photogrammetric evaluation, and the measurement of the angles was done using the NetCAD software (Ulusal CAD ve GIS Cozumleri AS, Ankara, Turkey). The photogrammetric evaluation was performed using 8–10 control points and 6 tie points (Figure 2) with the method of bundle block adjustment which is a mathematical technique (triangulation) that determines the position and orientation of each image as they existed at the time of image capture, determines the ground coordinates measured on overlap areas of multiple image, and minimizes the error associated with the imagery and image measurements. This is essentially a simultaneous triangulation performed on all observations. A 1 cm proximal excursion of the first point on the flexor tendon proximal to the A1 pulley was provided by the proximal pull of both finger flexors or thumb flexor of one finger at a time (Figure 3).

The angles between lines 1 and 2 were measured with

1. the TCL and the distal forearm fascia (FF) intact;
2. only the TCL incised;
3. the distal forearm fascia incised to the point 3 cm proximal from the most proximal part of the TCL in addition to the TCL.

The changes between the angles produced at all three conditions were compared to each other using SPSS program.

## 3. Results

The entrance angle of the flexor tendon to the A1 pulley was changed in all fingers but mostly in the third, fourth, and first fingers. This result was effective for both only TCL release and TCL and FF releases. However, it was more pronounced in the latter group.

**Figure 4:**
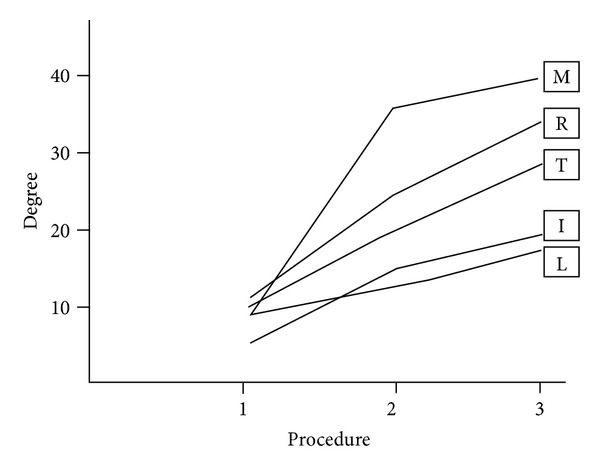
The graphical appearance of the increases of the flexor tendons' entrance angles to the A1 pulley according to the procedures 1, 2, and 3. The maximal increase is detected in the middle finger from procedure 1 to procedure 2. The minimal increase is detected in little finger. M: middle finger, R: ring finger, T: thumb, I: index finger, and L: little finger.

When TCL and FF were both intact, the mean of 10 A1 pulley entrance angles of flexor pollicis longus (FPL) tendons measured 10 degrees. The angle increased to mean 20 degrees by the TCL incision, and finally it measured 28 degrees by the addition of FF cut to TCL cut. For the index finger, the mean initial entrance angle was 4 degrees. When only the TCL was incised, the angle measured 16 degrees. The angle increased to 20 degrees with both TCL and FF cut. For the first procedure, the entrance angle of the tendon to A1 pulley was 8 degrees at the middle finger. With the release of TCL, it increased quite high and measured 35 degrees; however, the addition of FF release did not increase the angle significantly and it was 39 degrees with the cut of both anatomical structures (TCL, FF). In the ring finger, the angle was 12 degrees when TCL and FF were intact. The TCL cut increased the angle 2 times and it became 24 degrees. With the addition of FF cut, the angle measured 33 degrees. Finally, for the little finger, the entrance angle of the flexor tendon was 8 degrees with intact TCL and FF. The angle measured 11 and 13 degrees with only incised TCL and incised TCL, and FF, respectively (Table 1). From these results, it is seen that the maximal increase is detected in the middle finger from procedure 1 to procedure 2 and the minimal increase is detected in little finger (Figure 4).

Statistical analyses were performed by repeated measures of ANOVA (*P* < 0.05 accepted to be significant), and post hoc tests were performed by Bonferroni's adjusted paired *t*-test (*P* < 0.0017 accepted to be significant). According to these analyses, all our results were found to be statistically significant.

## 4. Discussion

CTS and TF are both important causes of occupational absenteeism and disability [5]. CTS is often associated with trigger digits. The incidence in various reports ranges from 0,2% to 22% [3, 5]. Phalen [7] reported a 5,2% incidence of TF in 654 CTS patients, and Harada et al. [4] reported an incidence of 11,5% in a group of 875 CTS patients. A more recent study by Kumar and Chakrabarti [8] reported that 43% of patients presenting with TF had also CTS. Additionally, some of these studies [3–5] pointed out that sometimes several weeks after carpal tunnel release, a trigger digit release becomes necessary. However, several studies reported the incidence of trigger finger which occurred after carpal tunnel release. Hombal and Owen [9] reported an incidence of 22% within one year of carpal tunnel release, and Harada et al. [4] reported an incidence of 5,9% which is considerably less than the previous one. The exact reason for the increased incidence of triggering after TCL release is unknown. Some possible explanations are edematous environment after surgery, an inflammatory process in the flexor tendons, and an increased friction at the A1 pulley due to the increased entrance angle of the tendons [9]. 

It can be thought that, in hands having either CTS or TF, there may be a pathologic condition of the patients' connective tissues which results in the other pathologic condition. However, studies reveal that histopathology of the connective tissue in CTS and TF is not the same. The pathologic finding in CTS is noninflammatory fibrosis of the subsynovial connective tissue with vascular hypertrophy and proliferation with obstruction and wall thickening [10] whereas pathology in TF is irregular connective tissue with small collagen fibres and abundant extracellular matrix containing chondroid matrix in the deep surface of the pulley [11]. As a result, we also think like the other authors who reported that these two conditions are not the results of the same pathologic condition of the connective tissue, so another mechanism should explain the concomitance of CTS and TF. Similarly systemic diseases like diabetes can be thought to be one of the ethiologies for concurrence of CTS and TF; however, Rottgers et al. [12], in their prospective clinical study, did not find that diabetes predisposed to the concomitant occurrence of the two conditions.

The TCL is thought to play a key role in the digital flexor pulley system [13]. It is suggested that biomechanically, the flexor tendons displace anteriorly after division of the TCL [14, 15], so a bowstringing is created which results in an increase in the entrance angle of the flexor tendons to the A1 pulley [9, 14, 16, 17], and this leads to a deterioration of the boundary lubrication mechanism of the tendon and pulley system [18]. Compressive forces increases and a fibrocartilagenous metaplasia of the connective tissue becomes possible at the side of increased compression which leads to triggering [19, 20]. Additionally, cadaver studies of Brown and Peimer [14] and Kiritsis and Kline [16] both reported an increased excursion of the tendons following TCL release. It can be speculated that combination of increased entrance angle and increased excursion can result in more fibrocartilagenous metaplasia due to friction between the tendon and the pulley. 

Conducting a cadaver study, we investigated the effect of TCL release with and without forearm fascia (FF) release on the occurrence of trigger digit. According to our literature search, this is the first cadaver study so far to show the relationship between carpal tunnel surgery and trigger finger.

To our knowledge, this study is also the first one to use the photogoniometric parameters to evaluate the tendons' positional behaviours after a surgical release in the hand. We also realised that although the effect of TCL release on the flexor tendons' positional behaviours was discussed in several previous reports [21], FF release had never been taken into account. Therefore, the measurement of A1 pulley entrance angles of flexor tendons was performed for all digits when TCL and FF were intact, TCL released, and both TCL and FF released. We saw that both TCL and TCL + FF releases increase the flexor tendons' entrance angles to the A1 pulley the increase in TCL + FF release is higher than only the TCL release, and all these differences are found to be statistically significant.

In conclusion, TCL and FF release may be a predisposing factor for the development of trigger finger by virtue of changing the entrance angle to the A1 pulley and consequently increase the friction in this anatomic area predisposing the triggering of the digit. Further prospective randomized control and cadaver studies are needed to confirm the effect of TCL and FF release on the development of trigger finger.

## Figures and Tables

**Table 1:** The entrance angles of tendons of all digits to A1 pulley according to the procedure.

	Thumb	Index	Middle	Ring	Little
Procedure 1	10°	4°	8°	12°	8°
Procedure 2	20°	16°	35°	24°	11°
Procedure 3	28°	20°	39°	33°	14°

Procedure 1: intact TCL and FF, Procedure 2: only the TCL incised, and Procedure 3: both TCL and FF incised. IF: index finger, MF: middle finger, RF: ring finger, and LF: little finger.
